# Intrauterine double-balloon tamponade vs gauze packing in the management of placenta previa: A multicentre randomized controlled trial

**DOI:** 10.1097/MD.0000000000019221

**Published:** 2020-02-14

**Authors:** Jing Wei, Yimin Dai, Zhiqun Wang, Ning Gu, Hongfang Ju, Youdi Xu, Biyun Xu, Yali Hu

**Affiliations:** a Department of Obstetrics and Gynecology, Nanjing Drum Tower Hospital, Affiliated to Nanjing Medical University, Nanjing; b Department of Obstetrics and Gynecology, Taizhou People's Hospital, Affiliated to Nantong University, Taizhou; c Department of Obstetrics and Gynecology, Nanjing First Hospital, Nanjing Medical University; d Department of Biomedical Statistics, Nanjing Drum Tower Hospital, Affiliated to Nanjing Medical University, Nanjing, China.

**Keywords:** placenta previa, postpartum hemorrhage, uterine balloon tamponade

## Abstract

**Background:**

To evaluate the effectiveness and safety of a newly designed intrauterine double-balloon catheter to arrest postpartum hemorrhage (PPH) following cesarean delivery (CD) for placenta previa.

**Methods:**

We conducted an open-label, multicenter randomized controlled trial in two referral centers and one general hospital. Women with continuous bleeding after placental delivery following CD for placenta previa, who failed to respond to uterotonics, suturing and uterine devascularization, and in the absence of suspected deeply invasive accreta were eligible subjects. Eligible subjects were randomized to receive intrauterine double-balloon catheter (n = 102) or gauze packing (n = 102). The main outcome was the rate of successful hemostasis without the need for additional surgical interventions. The secondary outcomes included the volume of blood loss during and after CD, the rate of PPH, incidence and amount of blood transfusion, hysterectomy, surgical complications, intensive care unit admission, need for re-laparotomy, length of hospital stay, and readmission.

**Results:**

The 224 participants were recruited before delivery, with 20 excluded (14 cases bleeding stopped after uterotonics and/or local myometrium sutures and 6 patients with placental increta). Finally, 102 women were assigned in catheter group and 102 others in gauze group. There was no difference in the rate of successful hemostasis in the catheter and gauze groups (93.1% vs 91.2%, *P* = .80). Compared with those in the gauze group, women in the catheter group showed significantly less blood loss within 24 hours postpartum (895 [612.3–1297.8] vs 1156 [882.5–1453.3] ml, *P* < .01), lower rate of PPH ≥1000 ml (42.2% vs 63.7%, *P* < .01). Accordingly, women in the catheter group had significantly less maternal adverse events such as postpartum anemia, puerperal morbidity, and postpartum pain.

**Conclusion:**

Uterine tamponade using a double-balloon catheter was as effective as gauze packing in hemostasis, and appeared to be superior in reducing postpartum blood loss and pain following CD for placenta previa. Using double-balloon catheter in managing PPH in this situation may be a preferable alternative to minimize maternal morbidity.

## Abstract 2

Supplemental Digital Content is available in the text

## 1 Introduction

Postpartum hemorrhage (PPH) following cesarean delivery (CD) for placenta previa is one of the leading causes of maternal morbidity and mortality.^[1]^ Approximately 10% of CD for placenta previa require intra- or post-operative blood transfusion, and ∼4% requiring peripartum hysterectomy.^[2–4]^ In China, with the cessation of the national one-child policy implemented in previous decades, the incidence of placenta previa has been increasing due to more mature mothers, previous spontaneous or elective pregnancy terminations, and most importantly previous CD.^[4]^ While correct placental location prenatally allows for individualized counseling and planning of the surgical management of the delivery,^[5]^ intrapartum and postpartum hemostasis at the lower uterine segment placenta site and related uterine atony remains a challenge.

Hemostasis at CD can be achieved with repeated doses of additional uterotonics, local suturing of the placental bed, uterine devascularization, or compression sutures such as the B-Lynch suture. In a resource-limited country such as China, uterine packing with gauze is a common practice to control PPH.^[6–7]^ In the 1980s, the idea of using an intrauterine balloon to produce a tamponade effect was introduced, and various types of balloon catheters have since been shown to produce similar successful rates of up to 91.5%.^[8]^ Owing to its simplicity, minimally invasive nature, and ease of application, balloon tamponade has become increasingly advocated as a first-line surgical intervention in the management of PPH to avoid more invasive interventions and hysterectomy.^[9–10]^ For placenta previa specifically, the use of the Bakri balloon, designed for the control bleeding from placenta previa, has decreased the rate of postpartum hysterectomy to less than a third of before.^[11]^ Nevertheless, the Bakri balloon can be associated with concealed bleeding, slide out of the uterine cavity easily, and even causing uterine perforation.^[12]^

**Figure 1:**
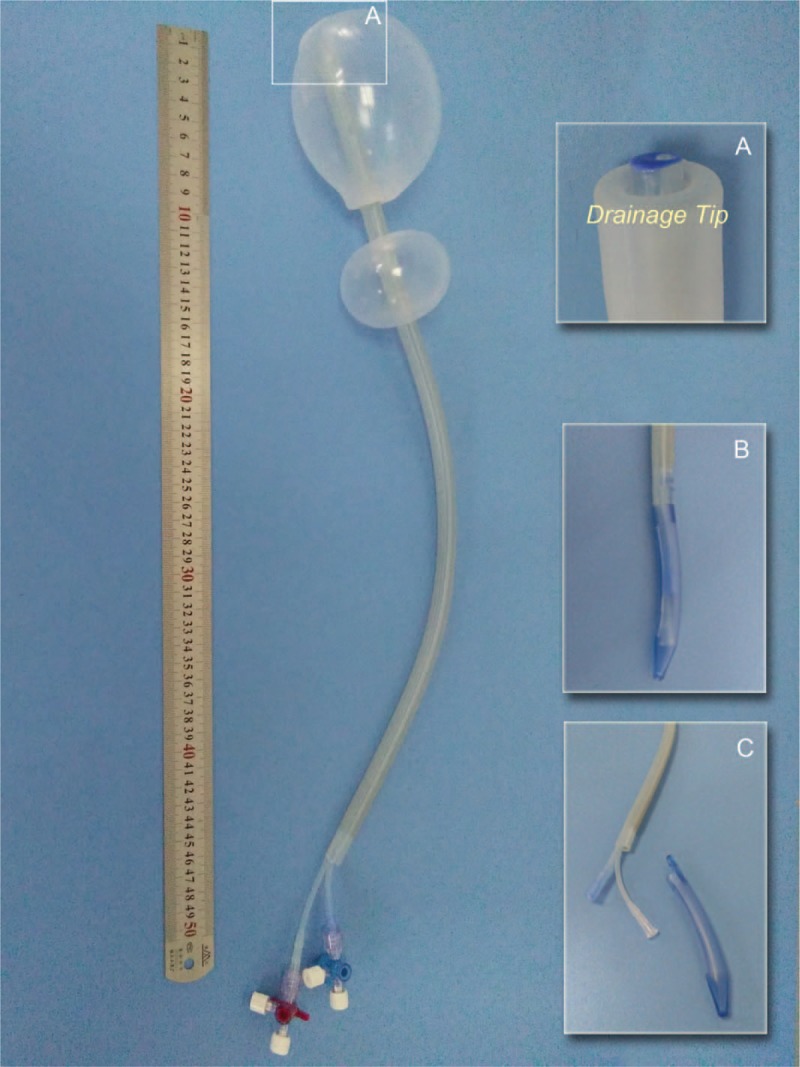
The new intrauterine double-balloon tamponade A. Drainage tip; B. Pen design at the end of balloon for CD; C. Drainage hole, upper and lower filling balloon.

Our group has designed an intrauterine double-balloon catheter with a wide-bore drainage (Fig. 1A). Then we developed an operation procedure for intrauterine tamponade using our catheter. In this randomized controlled study, we compared the efficacy of the two tamponade technologies for hemostasis in our protocols in participants with continuous bleeding who had been failed after treating with conservative measures following CD for placenta previa.

## 2 Materials and methods

### 2.1 Design of the cathetera

A double-balloon catheter for uterine tamponade was designed and developed in 2014. Following a pilot study,^[13]^ the catheter was modified and the final version is shown in Figure 1. The lower balloon was designed to generate direct pressure against the lower segment and internal os area which are the main bleeding sites in placenta previa. The upper balloon was designed to keep in the upper and middle uterine cavity for managing atony. The usual median total volume of the two balloons was 300 ml, which is comparable to the other balloons.^[13–16]^

During uterine closure, the lower balloon should be inflated first to pose local tamponade effect so as to reduce hemorrhage, and the partially inflated upper balloon provides room for visualization for the insertion of sutures to avoid catheter damaging. The gap between the two balloons avoids excessive pressure against the sutured incision from within which could lead to the suture cutting through the tissue after the balloons insufflated. The capsule caudal end facilitates trans-cervical insertion into the vagina (Fig. 1B, C). The stiffer large bore drainage tube design maintains patency by the implanted coil springs.

Since its introduction, the obstetricians participating in this study have been trained to apply this catheter successfully on many occasions. The development and adoption of this new balloon catheter in our institution is predominantly a cost issue, as it costs less than a third of the Bakri balloon catheter in China.

### 2.2 Trial design

This was a randomized controlled open-labelled study, conducted at three hospitals in Jiangsu province, China, between June 2015 and December 2017. The three hospitals have approximately 12,000 deliveries per annum, with PPH occurred in ∼5.3% women following CD. Among these hospitals, Nanjing Drum Tower Hospital has ∼7500 deliveries with 5.7% PPH, Taizhou People's Hospital, ∼3000 deliveries with 4.3% PPH, and Nanjing First Hospital, ∼1500 deliveries with 5.1% PPH every year. Before enrollment, the study was registered at Chinese Clinical Trial Registry (ChiCTR-ICR-15006467). All the participating staff had been trained to follow the trial protocol (see online supplementary materials).

### 2.3 Patient and public involvement

Pregnant women were not involved in the design or conduct of the trial. The results of the trial will not be disseminated directly to participants.

### 2.4 Inclusion and exclusion criteria

Eligibility criteria included:

1) placenta previa diagnosed before delivery,
2) age ≥18 years,
3) gestation ≥28 weeks of pregnancy, and
4) requiring CD.

At recruitment, all the women provided a written informed consent. A transvaginal ultrasound scan confirmed the placental location not earlier than 2 weeks prior to the planned delivery.

Exclusion criteria for the randomization included:

1) suspected deeply invasive placenta accreta,
2) fever above 38°C or chorioamnionitis at CD,
3) uterine malformation found at operation,
4) preoperational uterine artery embolisation,
5) uterine bleeding controlled following placenta delivery, after uterotonics and/or local myometrium sutures, or
6) the women had no desire to preserve the uterus.

### 2.5 Sample size and randomization

The estimated sample size was calculated based on the primary outcome of our previous pilot study and available data in the literature which showed that the rate of successful hemostasis was 97.7% by balloon tamponade, and 87.6% by gauze packing.^[17]^ Accordingly, 91 women per group were sufficient to show a difference of 10% in the successful rate as compared with the gauze with a 5% level of significance and a power of 80%. Considering the dropout rate of approximately 10%, finally, 102 women per arm were needed for randomization.

A computer-generated randomization code was produced and sequentially numbered. Block randomization with a 1:1 ratio for the double-balloon catheter or the gauze-roll group was set up with a block size of four. No stratification was performed. The numbers and codes were prepared and sealed in envelopes by a statistician of our institution before the trial. All the surgeons were blinded to the meaning of the numbers. During the CD, if tamponade method was decided, an envelope was opened by a nurse and revealed the allocation.

### 2.6 Intervention

CD was performed on lithotomy position and under spinal anesthesia. Uterine incision was encouraged to be made where feasible free of the placental edge. After fetal delivery and clamping of the umbilical cord, Carbetocin 100 μg (Ferring, St. Prex, Switzerland) was administered intravenously. The placenta was then removed by controlled cord traction. Manual separation would be performed in case of significant bleeding or suspected accreta. For controlling local bleeding from the placental bed, second-line uterotonic drugs (Hemabate 250 μg, Pharmacia & Upjohn Company, Kalamazoo, MI) and multiple interrupted sutures were used. Ligation of the uterine vessels would be performed based on the operator's judgement. If bleeding continued either from the placenta bed or due to atony, and regardless of the estimated blood loss at that point in time, tamponade method would be decided before any other interventions, and randomization was performed. For women allocated in the balloon catheter group (catheter group), the capsule caudal end of the device was inserted *via* the uterine incision through the cervix into and then retrieved from the vulval end of the vagina by an assistant. Both the lower small and the upper large balloons were kept in the uterine cavity and inflated initially with 50 ml and 100 ml of sterile normal saline respectively for the tamponade test. If successful, the uterine incision was closed in 2 layers with non-locking continuous suture, the balloons were then further inflated (lower balloon usually to 50–100 ml, and upper balloon to 150–350 ml) until the uterus was considered ‘firm’. At the end of the procedure, the drainage port of the balloon was connected to a graduated fluid collection bag to monitoring the amount of the bleeding.

For women in the gauze group, gauze rolls were packed in the cavity from the uterine fundus to the cervix with one end inserted into the vagina. Vaginal packing from posterior fornix to anterior fornix was placed afterwards to strengthen the compression to the lower uterine segment and maintain the position of the tamponade materials. If bleeding was continuous after tamponade, other additional measures were applied, including uterine artery embolization or hysterectomy. If bleeding was stopped, uterine and vaginal packing would be removed 12 to 24 hours later during day time. The Foley bladder catheter was kept in situ, and prophylactic antibiotic (cefazolin 1 g) was administered every 8 hours until the balloon or the gauze packing was removed. All the women were followed up to 6 weeks postpartum.

### 2.7 Outcomes

The primary outcome was the rate of successful hemostasis without the need for additional surgical interventions, involving artery embolization, hysterectomy, or replaced by another form of intrauterine tamponade.

The secondary outcomes included the volume of blood loss during and after CD, the rate of PPH of ≥1000 ml and ≥1500 ml, the incidence and amount of blood transfusion, hysterectomy, the duration of the entire operation, surgical complications (such as vascular, bladder and bowel injury, and others), intensive care unit admission, the need of re-laparotomy, length of hospital stay, and readmission after discharge.

Blood loss was measured according to the volume aspirated by suction, the weight of drapes, sanitary pads, gauze retrieved from uterine/vaginal cavities, and blood collected by the drainage bag in milliliters within 24 hours postpartum. Tolerability of the procedure by the women was assessed by VAS (visual analog scale) pain scores. Adverse events, such as double-balloon rupture or puncture, intrauterine material expelled into vagina, uterine perforation or rupture, and difficulty in removal of the tamponade material, were recorded throughout the trial.

### 2.8 Statistical analyses

Primary and secondary outcomes were analyzed on an intention-to-treat analysis. To assess the difference in two groups, *the student's t-test,* or *Man-Whitney U test* was used for continuous data depending on distribution, and the *Chi-squared test* or *Fisher exact test* where required was used for categorical data. Differences in proportions, medians (interquartile range) and mean (standard deviation) between the groups (with 95% confidence intervals) were also calculated, and adjusted for potential confounders where needed. A *P* value <.05 was considered statistically significant. Statistical analysis was performed using SPSS 20.0 (IBM, Armonk, NY).

## 3 Results

**Figure 2:**
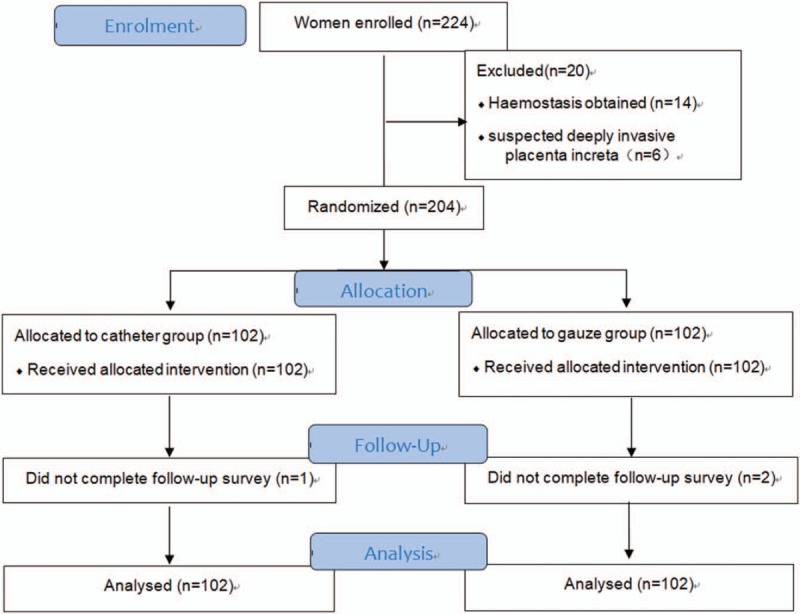
Participants flowchart through the trial.

During the study period, 224 patients with placenta previa were recruited, in whom 14 patients stopped bleeding after uterotonics and/or local myometrium sutures and 6 patients complicated with placental increta, so those patients were excluded (Fig. 2). One of the participating hospitals, which is a non-referral general hospital, managed to randomize 3 participants in 2 years due to the difficulty in recruitment. We included these cases in the study because all three were managed strictly according to the randomization and the protocol.

Baseline demographic and obstetric characteristics were comparable between the 2 groups (Table 1), and the proportion of complete placenta previa was similar. Successful hemostasis without need for additional surgical intervention was 93.1% (95/102) in the catheter group and 91.2% (93/102) in the gauze group (*P* = .80, Table 2). Before uterine tamponade, blood loss was similar between the 2 groups. Following tamponade, the median blood loss in the catheter group was 895 ml (interquartile range 612.3–1297.8), significantly lower than that in the gauze group (1156 ml [interquartile range 882.5–1453.3], *P* < .01). The frequency of PPH ≥1000 ml in the catheter group was significantly lower than that in the gauze group (42.2% vs 63.7%, *P* < .01). However, the frequency of PPH ≥1500 ml had no difference between the two groups (14.7% vs 19.6%, *P* = .35).

**Table 1:**
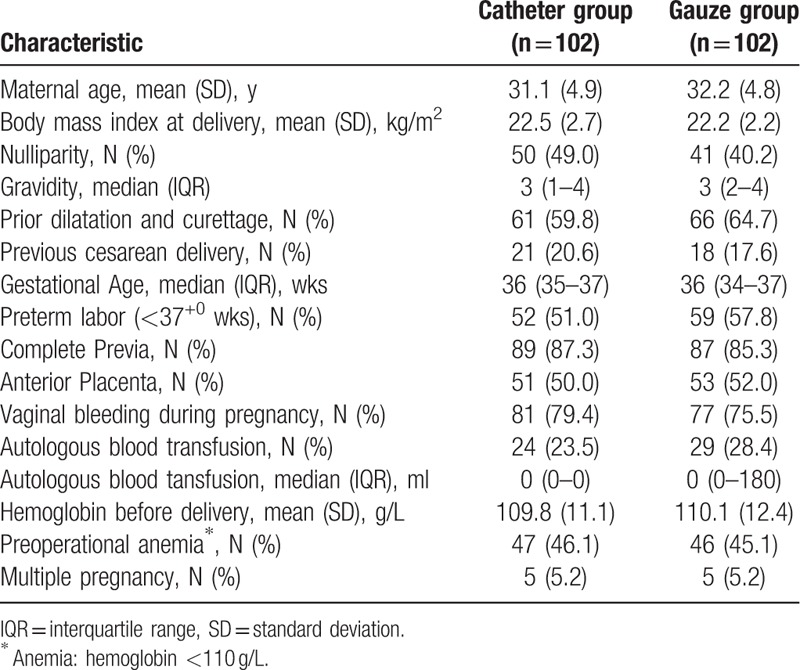
Demographic and clinical characteristics.

**Table 2:**
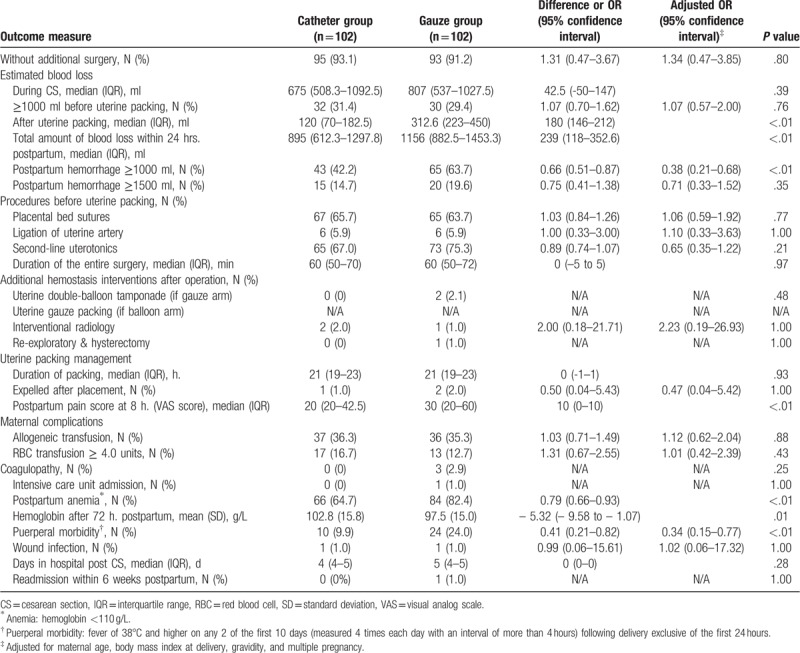
Surgery and maternal outcomes according to treatment allocation.

In the catheter group, 23 (22.5%) of the 102 women achieved hemostasis by filling the lower balloon only, and 74 others (72.5%) achieved hemostasis by filling both lower and upper balloons. The median volume infused into the lower and upper balloons was 100 ml (50–105 ml) and 200 ml (150–250 ml) respectively. The operation time was similar in both groups. Significantly lower VAS score at 8 hours postpartum was found in the catheter group (20 [interquartile range 20–42.5] vs 30 (interquartile range 20–60), *P* < .01). Both balloon and gauze were kept in the uterus for a median duration of 21 hours.

For maternal complications (Table 2), compared with the women in the gauze group, the women in the catheter group had lower postpartum anemia rate (64.7% vs 82.4%, *P* < 0.01), and lower puerperal morbidity (9.9% vs 24.0%, *P* < .01). The 2 groups had no significant difference in the other outcomes (Table 2).

Regarding the incidence of adverse events (see online supplementary table S1), 2 catheter balloons (2.1%) were inadvertently punctured by suturing needle, which were immediately replaced with new catheter balloons. Another catheter (1.0%) was expelled and passed into vagina, but the bleeding was subsequently controlled. Two cases (2.1%) in the gauze group had the gauze packing displaced into the vagina and led to massive bleeding; one was managed by balloon tamponade and the other was managed by artery embolization. No difficulty was encountered in removing the catheter, but, one case (1.0%) in the gauze group with displaced gauze had to have the gauze cut and pulled out under ultrasound guidance.

In all, 6 patients (2 in the catheter group, 4 in gauze group) failed to have bleeding arrested by tamponade. The details of those who required additional surgery are shown in online supplementary table S2. Among them, 3 were procedure-related (insufficient tamponade) which had to be managed during or shortly after the operation. In 3 others, late-onset hemorrhage happened immediately after removing the tamponade materials (1 in catheter group, 2 in gauze group), and the bleeding was successfully arrested by artery embolization, or repeated double-balloon tamponade or hysterectomy respectively. There was no maternal death in either group.

## 4 Discussion

Uterine packing is recommended in some guidelines despite a paucity of analytical studies,^[18–19]^ and it is widely used currently in both rich and poor areas in China as recommended by our national institution.^[20]^ As balloon catheter has become increasingly popular in the control of PPH, it is becoming the recommended treatment. In a country like China, the cost of commercially marketed balloon catheters such as the Bakri balloon has limited its clinical application. To date, no study was performed to compare the safety and efficacy of balloon tamponade with gauze packing. In the present study, we compared the safety and efficacy of our newly designed double-balloon catheter with traditional gauze packing in the management of placenta previa. Our results showed that, although either of balloon tamponade or gauze packing can achieve hemostasis in more than 90% of women, the women treated with balloon tamponade had less hemorrhage, lower incidence of PPH >1000 ml, decreased puerperal morbidity, and postpartum pain compared those treated with uterine gauze packing.

In previous reports, failure in the management of bleeding with balloon tamponade was attributed to damage or displacement of the balloon system.^[21–22]^ In this trial, 3.1% of the catheters were punctured or expelled spontaneous under our protocol in which vaginal packing was placed. Furthermore, no severe adverse event, such as genital tract perforation or rupture, was observed in the study.

The timing of balloon catheter insertion remains a controversy. Considering the efficiency and convenience of the balloons, lowering the threshold for using balloon tamponade has been advocated.^[23]^ A recent study found that severe blood loss before balloon tamponade increased the risks of procedure failure or treatment failure.^[19]^ In this study, balloon tamponade was used much earlier following second-line uterotonics and indicated placenta bed sutures. Despite the fact that 80% of the women had complete placenta previa, there was less maternal morbidity and need for further interventions. In line with previous studies,^[19,23]^ we considered that an earlier decision to use balloon tamponade could reduce the risk of PPH ≥1000 ml.

Our study was open-labelled randomized and implemented strictly following a pragmatic trial design. The measurement of blood loss was based on our previous and other researchers’ experience of using volume, weight as well as collecting bag. For accuracy, all commonly used drapes, absorbent pads, napkins, and gauze rolls were weighed before and after use, which enhanced the accuracy of blood loss estimation and the quality of the study.

There were some limitations in our work. First, placenta previa with suspected deep accreta overlying previous cesarean scar was excluded from this study because more invasive procedures and specialized management would be required in this scenario^[24]^ and these women should be studied separately. The exclusion of these women could have partially explained our high successful rate and the limited puerperal morbidity. Second, only 3 centers participate in this trial and therefore may not be generalizable outside of this region. Third, the inclusion criteria of ‘bleeding continued’ were based on personal judgment and thus not standard. And, we did not assess longer term complications due to our relatively short follow-up duration.

In conclusion, intrauterine double-balloon catheter is an effective substitute for traditional gauze packing in the management of PPH following CD for placenta previa, and is associated with less blood loss in the process. If a balloon is not available, uterine gauze packing is still an effective measure that can be utilized to avoid other invasive surgical procedures.

## Acknowledgments

All the double-balloon catheters used in the study were provided by the manufacturer free of charge. The manufacturer had no involvement in study design, data collection, data interpretation, or data analysis. We thank all the women and their families who consented to participate in the study. In addition, the study team thanks Professor TT Lao of the Chinese University of Hong Kong for help in revising the manuscript.

## Author contributions

JW and YD contributed equally to this article. JW was responsible for data collection and project management. YD was responsible for the procedures, interpreted results and manuscript preparation. BX prepared randomized number envelops and ran the statistical analysis. ZW, NG, HJ and YX were responsible for the procedures. YH made the contribution to the study design and reviewed the text. All the authors read and approved the final report.

## Supplementary Material

Supplemental Digital Content

## Supplementary Material

Supplemental Digital Content
