# Supplementary material for: Intrauterine double-balloon tamponade vs gauze packing in the management of placenta previa: A multicentre randomized controlled trial
Source: Medicine (Baltimore). 2020 Feb 14;99(7):e19221. doi: 10.1097/MD.0000000000019221 (PMC7035072; doi:10.1097/MD.0000000000019221)
Supplement: Supplemental Digital Content [file medi-99-e19221-s001.doc]

**Table S1 Adverse events o**f the two groups, N (%)

|  | Catheter group  (n=102) | Gauze group  (n=102) |
| --- | --- | --- |
| Inadvertent damage to the catheter | 2 (2.0) | 0(0) |
| Displaced into vagina | 1 (1.0) | 2(2.0) |
| Difficulty in removing | 0(0) | 1(1.0) |
| Uterine perforation | 0(0) | 0(0) |
| Uterine rupture | 0(0) | 0(0) |
