# Supplementary material for: Intrauterine double-balloon tamponade vs gauze packing in the management of placenta previa: A multicentre randomized controlled trial
Source: Medicine (Baltimore). 2020 Feb 14;99(7):e19221. doi: 10.1097/MD.0000000000019221 (PMC7035072; doi:10.1097/MD.0000000000019221)
Supplement: Supplemental Digital Content [file medi-99-e19221-s002.docx]

**Table S2 A summary of women details requiring second-line procedures**

| **Case** | **Intrauterine packing** | **Pregnancy history** | **Gestational age, wks** | **Risk factors** | **Intraoperative treatment** | **Timing of bleeding** | **Failure reason by audit** | **Second-line interventions** | **Total blood loss, ml** | **Operation duration, min** |
| --- | --- | --- | --- | --- | --- | --- | --- | --- | --- | --- |
| 1 | Double- balloon catheter | G2P0 | 38 | CPP  Placenta accreta | Placental bed sutures | Immediate after balloon catheter was moved  At 21 hrs. after CD | Late-onset hemorrhage | UAE | 1719 | 65 |
| 2 | Double- balloon catheter | G2P1 | 32 | CPP  Placenta increta | Placental bed sutures + Ligation of uterine arteries | During CD | Insufficient tamponade | UAE | 2300 | 66 |
| 3 | Gauze | G2P1 | 34 | CPP | Placental bed sutures | Immediate after CD | Insufficient packing | Intrauterine balloon catheter tamponade via vaginal | 1756 | 93 |
| 4 | Gauze | G3P1 | 38 | CPP  Placenta accreta | Placental bed sutures | 4 hrs. after CD | Gauze expelled into vagina related to insufficient packing | UAE | 3392 | 90 |
| 5 | Gauze | G2P1 | 33 | CPP  Multiple pregnancy, ART | Placental bed sutures | Immediate after gauze was moved  At 26 hrs. after CD | Late-onset  hemorrhage | Repeated tamponade by double-balloon catheter via vaginal | 1452 | 50 |
| 6 | Gauze | G3P1 | 28 | CPP  Placenta increta  Previous CD | Placental bed sutures | Immediate after gauze was moved  At 21 hrs. after CD | Late-onset  hemorrhage | Re-exploratory & hysterectomy | 6190 | 63 |

Abbreviation: CPP: complete placenta previa; CD: Cesarean delivery; UAE: uterine artery embolization; ART: Assisted reductive technology
